# Supplementary material for: Trends in the Development of Antibody-Drug Conjugates for Cancer Therapy
Source: Antibodies (Basel). 2023 Nov 3;12(4):72. doi: 10.3390/antib12040072 (PMC10660735; doi:10.3390/antib12040072)
Supplement: Supplementary file 1 [file antibodies-12-00072-s001.zip › antibodies-2638042-supplementary.pdf]

Trends in the development of antibody-drug conjugates for cancer therapy

Supplementary Materials

Table S1. List of ADC Research Articles

	Title	Linker	disease	Payload	Target antigen	Year
1	Peroxide-cleavable linkers for antibody–drug conjugates	cleavable	breast cancer	MMAE	HER2, pd-L1	2023
2	Antibody–Drug Conjugate Sacituzumab Govitecan Drives Efficient Tissue Penetration and Rapid Intracellular Drug Release	cleavable	gastric carcinoma	Topoisomerase 1 inhibitor	Trop-2	2023
3	An anti-EGFR antibody-drug conjugate overcomes resistance to HER2-targeted drugs	cleavable	breast cancer	DM1	HER2	2023
4	Targeting CD74 in B-cell non-Hodgkin lymphoma with the antibody-drug conjugate STRO-001	non-cleavable	b-cell non-Hodgkin's lymphoma	Maytansinoid	CD74	2023
5	Preclinical Evaluation of 9MW2821, a Site-Specific Monomethyl Auristatin E–based Antibody–Drug Conjugate for Treatment of Nectin-4–expressing Cancers	cleavable	urinary tract carcinoma	MMAE	Nectin-4	2023
6	SGN-CD228A Is an Investigational CD228-Directed Antibody–Drug Conjugate with Potent Antitumor Activity across a Wide Spectrum of Preclinical Solid Tumor Models	cleavable	melanoma, non-small cell lung cancer, breast cancer	MMAE	CD228	2023
7	Preclinical Profile of BYON3521 Predicts an Effective and Safe MET Antibody–Drug Conjugate	cleavable	gastric carcinoma	DNA-alkylating duocarmycin	c-MET	2023
8	Trastuzumab deruxtecan (DS-8201a), a HER2-targeting antibody–drug conjugate with topoisomerase I inhibitor payload, shows antitumor	cleavable	cervical cancer, ovarian cancer	Topoisomerase 1 inhibitor	HER2	2023

	activity in uterine and ovarian carcinosarcoma with HER2/neu expression					
9	Epigenetically upregulating TROP2 and SLFN11 enhances therapeutic efficacy of TROP2 antibody drug conjugate sacitizumab govitecan	cleavable	breast cancer	Topoisomerase 1 inhibitor	Trop-2	2023
10	Abstract ND01: ABBV-319: A first-in-class Glucocorticoid Receptor Modulator (GRM) agonist ADC for the treatment of B-cell malignancies	cleavable	b-cell malignancy	GRM	CD19	2023
11	A Novel Dual-Payload ADC for the Treatment of HER2+ Breast and Colon Cancer	cleavable	breast cancer, colorectal cancer	MMAE, DM1	HER2	2023
12	Tumor Site-Specific Cleavage Improves the Antitumor Efficacy of Antibody–Drug Conjugates	cleavable	breast cancer	MMAE	HER2	2023
13	Development of a MMAE-based antibody-drug conjugate targeting B7–H3 for glioblastoma	cleavable	non-small cell lung cancer	MMAE	B7-H3	2023
14	Abstract 6311: ABBV-400: An ADC delivering a novel topoisomerase 1 inhibitor to c-Met-positive solid tumors	cleavable	solid tumor	Topoisomerase 1 inhibitor	c-met	2023
15	A novel antibody-KSP inhibitor conjugate improves KSP inhibitor efficacy in vitro and in vivo	cleavable	breast cancer, gastric mucosal epithelial cancer	KSP inhibitor	HER2	2023
16	Preclinical development of a B7-H3-targeting ADC with a novel DNA topoisomerase I inhibitor for solid tumors	cleavable	lung cancer, glioma, prostate cancer	Topoisomerase 1 inhibitor	B7-H3	2023
17	Development of Triple-negative breast cancer (TNBC) syngeneic models and TROP2-directed antibody-drug conjugate (ADC) surrogate to model therapeutic combinations	cleavable	breast cancer	Topoisomerase 1 inhibitor	Trop-2	2023
18	Targeting CD44 Variant 5 with an Antibody–Drug Conjugate Is an Effective Therapeutic Strategy for Intrahepatic Cholangiocarcinoma	cleavable	cholangiocarcinoma	MMAE	CD44	2023
19	B7-H3 as a Therapeutic Target	cleavable	prostate cancer	Topoisomerase 1	B7-H3	2023

	in Advanced Prostate Cancer			inhibitor		
20	The first ADC bearing the ferroptosis inducer RSL3 as a payload with conservation of the fragile electrophilic warhead	cleavable	breast cancer	RSL3	HER2	2022
21	A FZD7-specific Antibody–Drug Conjugate Induces Ovarian Tumor Regression in Preclinical Models	cleavable	ovarian cancer	MMAE	FZD7	2022
22	Cellular-Resolution Imaging of Bystander Payload Tissue Penetration from Antibody-Drug Conjugates	cleavable	breast cancer	MMAE, Ahx maytansine, DM1, Calicheamicin D, PBD, Exatecan, DXd, SN 38, Calicheamicin G	HER2	2022
23	Preclinical Evaluation of IMGC936, a Next-Generation Maytansinoid-based Antibody–drug Conjugate Targeting ADAM9-expressing Tumors	cleavable	non-small cell lung cancer, pancreatic cancer, stomach cancer, breast cancer, ovarian cancer, colorectal cancer	DM21C	ADAM9	2022
24	DS-7300a, a DNA Topoisomerase I Inhibitor, DXd-Based Antibody–Drug Conjugate Targeting B7-H3, Exerts Potent Antitumor Activities in Preclinical Models	cleavable	small cell lung cancer, esophageal cancer, endometrial cancer, prostate cancer, breast cancer	DXd	B7-H3	2022
25	Overcoming Resistance to Anti–Nectin-4 Antibody-Drug Conjugate	cleavable	breast cancer	MMAE	Nectin-4	2022
26	EGFR Inhibition Enhances the Cellular Uptake and Antitumor-Activity of the HER3 Antibody–Drug Conjugate HER3–DXd	cleavable	non-small cell lung cancer	MMAE	HER3	2022
27	A CD45-targeted antibody-drug conjugate successfully conditions for allogeneic hematopoietic stem cell transplantation in mice	cleavable	blood cancer	PBD	CD45	2022
28	Monomethyl auristatin antibody and peptide drug conjugates for trimodal cancer chemo-radio-immunotherapy	cleavable	head and neck cancer, lung cancer, melanoma, colorectal cancer	MMAE	HER3	2022

29	CUB Domain-Containing Protein 1 (CDCP1) is a rational target for the development of imaging tracers and antibody-drug conjugates for cancer detection and therapy	cleavable	kidney cancer, colorectal cancer, lung cancer, ovarian cancer, pancreatic cancer, prostate cancer	MMAE	CDCP1	2022
30	Targeting LRRC15 Inhibits Metastatic Dissemination of Ovarian Cancer	cleavable	ovarian cancer	MMAE	LRRC15	2022
31	ABBV-011, A Novel, Calicheamicin-Based Antibody–Drug Conjugate, Targets SEZ6 to Eradicate Small Cell Lung Cancer Tumors	cleavable	small cell lung cancer	Calicheamicin	SEZ6	2022
32	Exploiting LRRC15 as a Novel Therapeutic Target in Cancer	cleavable	pancreatic cancer, breast cancer, sarcoma,ovarian cancer	MMAE	LRRC15	2022
33	Patritumab deruxtecan (HER3-DXd), a novel HER3 directed antibody drug conjugate, exhibits in vitro activity against breast cancer cells expressing HER3 mutations with and without HER2 overexpression	cleavable	breast cancer	DXd	HER3	2022
34	EpCAM- and EGFR-Specific Antibody Drug Conjugates for Triple-Negative Breast Cancer Treatment	cleavable	breast cancer	MMAE	EGFR, EpCAM	2022
35	A Novel NAMPT Inhibitor-Based Antibody–Drug Conjugate Payload Class for Cancer Therapy	cleavable, non-cleavable	colorectal cancer, breast cancer, ovarian cancer	NAMPTi	LYPD3, HER2, B7-H3	2022
36	Antibody-Drug Conjugate Targeting c-Kit for the Treatment of Small Cell Lung Cancer	non-cleavable	small cell lung cancer	DM1	c-Kit	2022
37	Antibody drug conjugates, targeting cancer-expressed EGFR, exhibit potent and specific antitumor activity	cleavable, non-cleavable	epithelial cancer, non-small cell lung cancer, colorectal cancer, head and neck cancer	MMAE, DXd1, SN-38, Tesirine, DM1	EGFR	2022
38	Therapeutic effect of a MUC1-specific monoclonal antibody-drug conjugates against pancreatic cancer model	cleavable	pancreatic cancer	MMAE	MUC1	2022

39	A Novel Antibody-Drug Conjugate Targeting Nectin-2 Suppresses Ovarian Cancer Progression in Mouse Xenograft Models	non-cleavable	ovarian cancer	DM1	Nectin-2	2022
40	A novel anti-c-Kit antibody–drug conjugate to treat wild-type and activating-mutant c-Kit-positive tumors	non-cleavable	gastrointestinal stromal tumors, small cell lung cancer	DM1	c-Kit	2022
41	HER2-Targeted Antibody–Drug Conjugates Display Potent Antitumor Activities in Preclinical Extramammary Paget's Disease Models: In Vivo and Immunohistochemical Analyses	cleavable, non-cleavable	Paget's disease of the breast	DXd, DM1	HER2	2022
42	EGFR binding Fc domain-drug conjugates: stable and highly potent cytotoxic molecules mediate selective cell killing	cleavable	small cell lung cancer, head and neck cancer, colon cancer, pancreatic cancer	MMAE	EGFR	2022
43	Fcγ Receptor-Dependent Internalization and Off-Target Cytotoxicity of Antibody-Drug Conjugate Aggregates	cleavable, non-cleavable	breast cancer	DXd, DM1	HER2	2022
44	Lipolysis-stimulated lipoprotein receptor-targeted antibody-drug conjugate demonstrates potent antitumor activity against epithelial ovarian cancer	cleavable	ovarian cancer	MMAE	LSR	2022
45	hysicochemical and biological impact of metal-catalyzed oxidation of IgG1 monoclonal antibodies and antibody-drug conjugates via reactive oxygen species	non-cleavable, cleavable	breast cancer, stomach cancer, ovarian cancer, non-small cell lung cancer, thyroid cancer	DM1, MMAE	HER2, NaPi2b	2022
46	All-in-one disulfide bridging enables the generation of antibody conjugates with modular cargo loading	cleavable	breast cancer	MMAE	HER2	2022
47	Biological Evaluation of Maytansinoid-Based Site-Specific Antibody-Drug Conjugate Produced by Fully Chemical Conjugation Approach: AJICAP®	cleavable	breast cancer	Maytansinoid	HER2	2022
48	High-throughput membrane-	cleavable	esophageal	MMAE	PIEZO1	2022

	anchored proteome screening reveals PIEZO1 as a promising antibody-drug target for human esophageal squamous cell carcinoma		cancer			
49	Evaluation of the DLL3-targeting Antibody–Drug Conjugate Rovalpituzumab Tesirine in Preclinical Models of Neuroblastoma	cleavable	neuroblastoma	PBD dimer	DLL3	2022
50	Preclinical Development of ADCT-601, a Novel Pyrrolobenzodiazepine Dimer-based Antibody–drug Conjugate Targeting AXL-expressing Cancers	cleavable	non-small cell lung cancer, Acute myeloid leukemia, melanoma, ovarian cancer, kidney cancer, pancreatic cancer, esophageal cancer,	PBD dimer	AXL	2022
51	Enapotamab Vedotin, an AXL-Specific Antibody-Drug Conjugate, Demonstrates Antitumor Efficacy in Patient-Derived Xenograft Models of Soft Tissue Sarcoma	cleavable	melanoma, pancreatic cancer, cervical cancer	MMAE	AXL	2022
52	GSK3 inhibitor enhances gemtuzumab ozogamicin-induced apoptosis in primary human leukemia cells by overcoming multiple mechanisms of resistance	cleavable	breast cancer	Calicheamicin	CD33	2022
53	Discovery of ABBV-3373, an Anti-TNF Glucocorticoid Receptor Modulator Immunology Antibody Drug Conjugate	cleavable	breast cancer, non-small cell lung cancer	GRM	α-TNF	2022
54	Antibody-drug conjugates with dual payloads for combating breast tumor heterogeneity and drug resistance	cleavable	breast cancer	MMAE, MMAF	HER2	2021
55	Datopotamab Deruxtecan, a Novel TROP2-directed Antibody-drug Conjugate, Demonstrates Potent Antitumor Activity by Efficient Drug Delivery to Tumor Cells	cleavable	melanoma	DXd	Trop-2	2021
56	Improving Antibody-Tubulysin Conjugates through Linker	cleavable	leukemia	Tubulysin M	CD30	2021

	Chemistry and Site-Specific Conjugation					
57	Calicheamicin Antibody–Drug Conjugates with Improved Properties	not used	Acute myeloid leukemia	Calicheamicin	CD30, CD22	2021
58	Tandem-Cleavage Linkers Improve the In Vivo Stability and Tolerability of Antibody–Drug Conjugates	cleavable	chronic lymphocytic leukemia	MMAE	CD79b	2021
59	Novel antibody-drug conjugate with UV-controlled cleavage mechanism for cytotoxin release	cleavable	breast cancer, gastric carcinoma	MMAE	HER2	2021
60	Exatecan Antibody Drug Conjugates Based on a Hydrophilic Polysarcosine Drug-Linker Platform	cleavable	breast cancer, stomach cancer	Topoisomerase 1 inhibitor	HER2	2021
61	Preclinical Antitumor Activity and Biodistribution of a Novel Anti-GCC Antibody–Drug Conjugate in Patient-derived Xenografts	cleavable	colorectal cancer, esophageal cancer, stomach cancer, pancreatic cancer	IGN dimer	Guanylyl cyclase C	2020
62	LILRB4-targeting Antibody–Drug Conjugates for the Treatment of Acute Myeloid Leukemia	cleavable	Acute myeloid leukemia	MMAF	LILRB4	2020
63	A Novel Antibody-Drug Conjugate (ADC) Delivering a DNA Mono-Alkylating Payload to Chondroitin Sulfate Proteoglycan (CSPG4)-Expressing Melanoma	cleavable	melanoma	Pyrridinobenzodiazepine	CSPG4	2020
64	Targeting Multiple EGFR-expressing Tumors with a Highly Potent Tumor-selective Antibody–Drug Conjugate	cleavable	small cell lung cancer, head and neck cancer, colon cancer, pancreatic cancer	PBD dimer	EGFR	2020
65	Depatuxizumab Mafodotin (ABT-414)-induced Glioblastoma Cell Death Requires EGFR Overexpression, but not EGFRY1068 Phosphorylation	non-cleavable	glioblastoma	MMAF	EGFR	2020
66	Antibody-Drug Conjugate Using Ionized Cys-Linker-MMAE as the Potent Payload Shows Optimal Therapeutic Safety	non-cleavable	breast cancer	MMAE	HER2	2020
67	On the use of DNA as a linker	cleavable	breast cancer	MMAE	HER2	2020

	in antibody-drug conjugates: synthesis, stability and in vitro potency					
68	TR1801-ADC: a highly potent cMet antibody-drug conjugate with high activity in patient-derived xenograft models of solid tumors	cleavable	stomach cancer, colorectal cancer, head and neck cancer	Pyrrolobenzodiazepine	c-Met	2019
69	A HER2-Targeting Antibody–Drug Conjugate, Trastuzumab Deruxtecan (DS-8201a), Enhances Antitumor Immunity in a Mouse Model	cleavable	breast cancer, stomach cancer, esophageal cancer, colorectal cancer, salivary cancer	Topoisomerase 1 inhibitor	HER2	2018
70	Preparation of an Ultrahigh-DAR PDL1 monoclonal antibody-polymeric-SN38 conjugate for precise colon cancer therapy	cleavable	colorectal cancer	SN-38	PD-L1	2023
71	Preclinical Efficacy of the Antibody–Drug Conjugate CLDN6–23-ADC for the Treatment of CLDN6-Positive Solid Tumors	cleavable	ovarian cancer	CLDN6	MMAE	2023

Table S2. List of ADC clinical trials

No.	Clinical Trials	ADC / Linker / Payload	Antigen	Phase	Year
1	A Phase I, Open-label, Multi-center, First in Human, Dose Escalation and Expansion Study to Assess the Safety, Tolerability, Efficacy and Pharmacokinetics of MRG002 in Patients With HER2 Positive Advanced Solid Tumors	MRG002/Val-Cit/MMAE	HER2	1	2018
2	A Randomized, Controlled, Multi-center	RC48-ADC/Val-Cit/MMAE	HER2	2,3	2018

	Phase II Clinical Study to Evaluate the Efficacy and Safety of Recombinant Humanized Anti-HER2 Monoclonal Antibody-MMAE Conjugate for Injection in the Treatment of HER2-positive Locally Advanced or Metastatic Breast Cancer and Phase III Clinical Study to Evaluate the Efficacy and Safety of Recombinant Humanized Anti-HER2 Monoclonal Antibody-MMAE Conjugate for Injection in the Treatment of HER2-positive Advanced Breast With Liver Metastases				
3	A PHASE 3, MULTICENTER, RANDOMIZED, OPEN-LABEL, ACTIVE-CONTROLLED TRIAL OF DS-8201A, AN ANTI-HER2-ANTIBODY DRUG CONJUGATE (ADC), VERSUS TREATMENT OF PHYSICIAN'S CHOICE FOR HER2-LOW, UNRESECTABLE AND/OR METASTATIC BREAST CANCER SUBJECTS	DS-8201/Tetrapeptide-based/Topoisomerase I inhibitor	HER2	3	2019
4	A Phase I, Open-label, Dose Escalation Clinical Trial to Assess the Safety, Efficacy, Tolerability and Pharmacokinetics of the Recombinant Humanized Anti-PD1 Monoclonal Antiody (JS001) in Combination With Recombinant Humanized Anti-HER2 Monoclonal Antibody-MMAE Conjugate (RC48-ADC) in Treatment of HER2-Positive Advanced Malignant Solid Tumors	RC48-ADC/Val-Cit/MMAE	HER2	1	2020
5	A Phase 1b/2 Multicenter, Open-label, Dose-escalation and Dose expansion Study to Evaluate the Safety, Tolerability, Pharmacokinetics, Immunogenicity, and Antitumor Activity of Trastuzumab Deruxtec..	DS-1062/Tetrapeptide-based/Topoisomerase I inhibitor	HER2	1,2	2020
6	A Phase 2, Multicenter, Open-label Study to Evaluate the Efficacy and Safety of Trastuzumab Deruxtecan (T-DXd, DS-8201a) for the Treatment of Selected HER2 Expressing Tumors (DESTINY-PanTumor02)	DS-8201/Tetrapeptide-based/Topoisomerase I inhibitor	HER2	2	2020
7	An Open-label, Single-arm, Multi-center, Phase II Study of RC48-ADC in Subjects With HER2 Overexpressed Locally Advanced or Metastatic Biliary Tract Cancer (BTC) Who Have Failed First-line Chemotherapy	RC48-ADC/Val-Cit/MMAE	HER2	2	2020
8	Randomized, Controlled, Multicenter Phase III Clinical Study Evaluating the Efficacy and Safety of RC48-ADC for the Treatment of Locally Advanced or Metastatic Breast Cancer With Low Expression of HER2	RC48-ADC/Val-Cit/MMAE	HER2	3	2020
9	Safety and unique pharmacokinetic profile	ARX788/pAcF/MMAF	HER2	1	2021

	of ARX788, a site-specific ADC, in heavily pretreated patients with HER2-overexpresing solid tumors: Results from two phase 1 clinical trials.				
10	An Open-Label, Multi-center Phase I/II Dose Escalation and Expansion Study to Assess the Safety, Efficacy and Pharmacokinetics of MRG002 in Patients With HER2-Positive Advanced Solid Tumors and Locally Advanced or Metastatic Gastric/Gastroesophageal Junction (GEJ) Cancer	MRG002/Val-Cit/MMAE	HER2	1,2	2021
11	A Single-arm, Single-center, Open Phase Ⅱa Clinical Study of RC48-ADC in the Treatment of HER2-positive Advanced Melanoma	RC48-ADC/Val-Cit/MMAE	HER2	2	2021
12	An Open, Multi-cohort, Multi-center Phase II Basket Clinical Study of RC48-ADC Monotherapy in the Treatment of HER2-expressing (HER2-positive and HER2 Low Expression) Gynecological Malignancies	RC48-ADC/Val-Cit/MMAE	HER2	2	2021
13	A Clinical Study of RC48-ADC Combined With Triplizumab as Neoadjuvant Therapy Before Radical Resection of Myometrial Invasive Bladder Cancer	RC48-ADC/Val-Cit/MMAE	HER2	2	2021
14	Antibody-Drug Conjugate Combined With Hypofractionated Radiotherapy, PD-1/PD-L1 Inhibitor Sequential GM-CSF and IL-2 for the Treatment Advanced Refractory Solid Tumors With HER-2 Positive(PRaG3.0)	RC48-ADC/Val-Cit/MMAE	HER2	2	2021
15	Phase II Clinical Study to Evaluate the Efficacy and Safety of Recombinant Humanized Anti-HER2 Monoclonal Antibody-MMAE Coupling Agent (RC48-ADC) for Neoadjuvant Treatment of Breast Cancer With Positive HER2 Expression (Seraph)	RC48-ADC/Val-Cit/MMAE	HER2	2	2021
16	An Open-label, Single-arm, Multi-center, Phase II Clinical Study of MRG002 in the Treatment of Patients With HER2-positive Unresectable Locally Advanced or Metastatic Urothelium Cancer	MRG002/Val-Cit/MMAE	HER2	2	2021
17	An Open-label, Single-arm, Multi-center, Phase II Clinical Study of MRG002 in the Treatment of Patients With HER2-positive Unresectable, Locally Advanced or Metastatic Biliary Tract Cancer	MRG002/Val-Cit/MMAE	HER2	2	2021
18	A Multicenter, Non-randomized, Open-label Phase II Clinical Study to Evaluate the Efficacy and Safety of MRG002 in the	MRG002/Val-Cit/MMAE	HER2	2	2021

	Treatment of Patients With HER2-low Locally Advanced or Metastatic Breast Cancer (BC)				
19	An Open-label, Multi-center, Non-randomized Phase II Clinical Study to Evaluate the Efficacy and Safety of MRG002 in Patients With HER2-mutated Unresectable/Metastatic Non-small Cell Lung Cancer (NSCLC).	MRG002/Val-Cit/MMAE	HER2	2	2021
20	A Phase 2, Multicenter, Randomized, Study of Trastuzumab Deruxtecan in Subjects with HER2-overexpressing Locally Advanced, Unresectable or Metastatic Colorectal Cancer (DESTINY-CRC02)	DS-8201/Tetrapeptide-based/Topoisomerase I inhibitor	HER2	2	2021
21	A Phase 2, multicenter, randomized study of trastuzumab deruxtecan in subjects with HER2-mutated metastatic Non-Small Cell Lung Cancer (NSCLC) [DESTINY-Lung02]	DS-8201/Tetrapeptide-based/Topoisomerase I inhibitor	HER2	2	2021
22	A Study of MRG002 in the Treatment of Patients With HER2-positive Unresectable Locally Advanced or Metastatic Breast Cancer	MRG002/Val-Cit/MMAE	HER2	2,3	2021
23	Randomized, Controlled, Multicenter Phase III Clinical Study Evaluating the Efficacy and Safety of RC48-ADC for the Treatment of Locally Advanced or Metastatic Gastric Cancer With HER2-overexpression	RC48-ADC/Val-Cit/MMAE	HER2	3	2021
24	A RANDOMIZED, MULTICENTER, DOUBLEBLIND, PLACEBO-CONTROLLED PHASE III STUDY OF THE EFFICACY AND SAFETY OF TRASTUZUMAB EMTANSINE IN COMBINATION WITH ATEZOLIZUMAB OR PLACEBO IN PATIENTS WITH HER2-POSI...	T-DM1/SMCC/DM1	HER2	3	2021
25	Phase III Study of Trastuzumab Deruxtecan (T-DXd) with or without Pertuzumab versus Taxane, Trastuzumab and Pertuzumab in HER2-positive, First-line Metastatic Breast Cancer (DESTINY-Breast09)	DS-8201/Tetrapeptide-based/Topoisomerase I inhibitor	HER2	3	2021
26	A Phase 3 Open-label Trial of Neoadjuvant Trastuzumab Deruxtecan (TDXd) Monotherapy or T-DXd followed by THP Compared to ddAC-THP in Participants with High-risk HER2-positive Early-stage Breast Can...	DS-8201/Tetrapeptide-based/Topoisomerase I inhibitor	HER2	3	2021
27	An Open-Label, Multinational, Multicenter, Phase 3b/4 Study of Trastuzumab Deruxtecan in Patients With or Without Baseline Brain Metastasis With Previously Treated Advanced/Metastatic HER2-Positive Breast Cancer (DESTINY-Breast12)	DS-8201/Tetrapeptide-based/Topoisomerase I inhibitor	HER2	3b/4	2021

28	A Phase I Study to Evaluate the Safety, Tolerability, Pharmacokinetics and Efficacy of FDA022-BB05 in Subjects With Advanced Solid Malignant Tumors	FDA022-BB05/Unknown/Topoisomerase I inhibitor	HER2	1	2022
29	460MO Preliminary results from a phase I study using the bispecific, human epidermal growth factor 2 (HER2)-targeting antibody-drug conjugate (ADC) zanidatamab zovodotin (ZW49) in solid cancers	ZW49/Val-Cit/N-acyl sulfonamide auristatin	HER2	1	2022
30	A Multicenter, Open Label , Single Arm , Phase Ib/II Study to Evaluate the Effect and Safety of RC48-ADC Combined With S-1 for First-line Treatment of Advanced Gastroesophageal Adenocarcinoma With Moderate Expression of HER2	RC48-ADC/Val-Cit/MMAE	HER2	1,2	2022
31	An Open-label, Multi-center, Phase I/II Dose Escalation and Expansion Study to Evaluate the Safety, Tolerability, Pharmacokinetics and Preliminary Efficacy of MRG002 in Combination With HX008 in Patients With HER2-expressed Advanced Malignant Solid Tumors.	MRG002/Val-Cit/MMAE	HER2	1,2	2022
32	A Phase 1/2, First-in-Human, Open-Label, Dose-Escalation and Expansion Study of IMGC936-0901 (Anti-ADAM9 Antibody Drug Conjugate) in Patients with Advanced Solid Tumors	IMGC936/Tripeptide linker/DM21	HER2	1,2	2022
33	RC48-ADC in Combination With Envolizumab for the First-line Treatment of Locally Advanced or Metastatic Biliary Tract Cancer With Positive HER-2: A Prospective, Single-arm Phase II Trial.	RC48-ADC/Val-Cit/MMAE	HER2	2	2022
34	An Open-label, Single-arm Study to Evaluate the Efficacy and Safety of RC48-ADC Combined With JS001 in Postoperative Adjuvant Treatment of HER2-positive Upper Tract Urothelial Carcinoma (UTUC)	RC48-ADC/Val-Cit/MMAE	HER2	2	2022
35	A Multicenter, Single Arm Phase II Clinical Study Evaluating the Efficacy and Safety of RC48-ADC for the Treatment of HER2-expression Metastatic Breast Cancer With Abnormal Activation of PAM Pathway	RC48-ADC/Val-Cit/MMAE	HER2	2	2022
36	An Open-label, Single-arm, Multi-center, Phase II Study to Evaluate the Efficacy and Safety of RC48-ADC Combined With JS001 in Perioperative Treatment of Muscle-Invasive Bladder Cancer	RC48-ADC/Val-Cit/MMAE	HER2	2	2022
37	An Open-label, Single-arm, Multicenter Phase II Clinical Study : Evaluating RC48-	RC48-ADC/Val-Cit/MMAE	HER2	2	2022

	ADC Combined With Triplizumab in the Neoadjuvant Treatment of Her2-positive Muscle-invasive Bladder Cancer				
38	Phase II Clinical Study to Assess the Efficacy and Safety of Recombinant Humanized Anti-HER2 Monoclonal Antibody MMAE Coupling Agent in Treating Patients With Locally Advanced or Metastatic Salivary Gland Tumors Expressing HER2	RC48-ADC/Val-Cit/MMAE	HER2	2	2022
39	An Open-label, Multi-center, Single-arm Phase II Clinical Study to Evaluate the Efficacy and Safety of MRG002 in Advanced HER-2 Positive Breast Cancer Patients Previously Treated With Trastuzumab and TKIs (Magic-009)	MRG002/Val-Cit/MMAE	HER2	2	2022
40	A Phase 2 Multi-Cohort, Open-Label, Multi-Center Clinical Study Evaluating the Efficacy and Safety of Disitamab Vedotin (RC48-ADC) Alone or in Combination With Pembrolizumab in Subjects With Locally-Advanced Unresectable or Metastatic Urothelial Carcinoma That Expresses HER2	RC48-ADC/Val-Cit/MMAE	HER2	2	2022
41	Disitamab Vedotin Combined With Tislelizumab, Low-dose Capecitabine and Celecoxib as Salvage Therapy for HER2-positive Metastatic Colorectal Cancer: a Phase II Trial (DETECT)	RC48-ADC/Val-Cit/MMAE	HER2	2	2022
42	A Single-arm, Open Clinical Trial of Efficacy and Safety of Tislelizumab in Combination With Disitamab-vedotin as Neoadjuvant Therapy for HER2-positive High-risk Upper Tract Urothelial Carcinoma (UTUC)	RC48-ADC/Val-Cit/MMAE	HER2	2	2022
43	An Open-label, Multi-center, Phase II Clinical Study to Evaluate the Safety, Efficacy and Pharmacokinetics of MRG002 in Patients With HER2-positive/HER2-low Locally Advanced or Metastatic Gastric/ Gastroesophageal Junction Cancer.	MRG002/Val-Cit/MMAE	HER2	2	2022
44	A Single-arm, Prospective, Open-label Clinical Study of Tislelizumab Combined With DisitamabVedotin and Pyrotinib Maleate in HER2-positive or Mutated Advanced Colorectal Cancer Who Failed Standard Therapy	RC48-ADC/Val-Cit/MMAE	HER2	2	2022
45	A Global, Phase 2 Study of ARX788 in HER2-positive Metastatic Breast Cancer Patients Whose Disease is Resistant or Refractory to T-DM1, and/or T-DXd, and/or Tucatinib-containing Regimens	ARX788/pAcF/MMAF	HER2	2	2022

46	A PHASE 2 MULTI COHORT, OPEN LABEL, MULTI CENTER CLINICAL STUDY EVALUATING THE EFFICACY AND SAFETY OF DISITAMAB VEDOTIN (RC48 ADC) IN SUBJECTS WITH HER2 EXPRESSING LOCALLY ADVANCED UNRESECTABLE OR METASTATIC UROTHELIAL CARCINOMA	RC48-ADC/Val-Cit/MMAE	HER2	2	2022
47	A multiple center, open-label, single-arm, phase II clinical trial of MRG002, an HER2-targeted antibody-drug conjugate, in patients with HER2-low expressing advanced or metastatic breast cancer.	MRG002/Val-Cit/MMAE	HER2	2	2022
48	A Open-Label, Multicenter, Randomised, Controlled Phase 3 Study of RC48-ADC Plus Toripalimab Versus Chemotherapy Alone in Previously Untreated Unresectable Locally Advanced or Metastatic Urothelial Carcinoma With HER2-Expressing	RC48-ADC/Val-Cit/MMAE	HER2	3	2022
49	A Single-arm, Open, Multicenter Phase I Study to Evaluate the Safety, Tolerability, Pharmacokinetic Profile, and Efficacy of the KM501 Double-antibody ADC in Subjects With Advanced Solid Tumors That Express, Amplify, or Mutate HER2	KM501/Unknown/Unknown	HER2	1	2023
50	A Phase 1 Dose Escalation Trial to Determine the Safety, Tolerance, Maximum Tolerated Dose, and Preliminary Antineoplastic Activity of IKS014, a HER2-Targeting Antibody Drug Conjugate (ADC), in Participants With Advanced HER2+ Solid Tumors	IKS014/Glucuronide-trigger/MMAF	HER2	1	2023
51	Evaluate the Safety, Tolerability, Pharmacokinetic Characteristics, and Preliminary Efficacy of Disitamab Vedotin Intravenously Combined With Radiotherapy in the Treatment of Locally Advanced Solid Tumors With HER2 Expression Phase 1 Study	RC48-ADC/Val-Cit/MMAE	HER2	1	2023
52	An Open-label, Single-center, Phase Ib/II Study to Evaluate the Safety, Efficacy and Pharmacokinetics of RC48-ADC Combined With Pyrotinib in Local Advanced or Metastasis NSCLC With HER2 Mutation	RC48-ADC/Val-Cit/MMAE	HER2	1,2	2023
53	RC48-ADC Combined With Bevacizumab in HER2-positive Advanced Colorectal Cancer : a Single-arm, Non-randomized, Single-center Trial	RC48-ADC/Val-Cit/MMAE	HER2	2	2023
54	Prospective, Open, Single-center Clinical Study of the Combination of RC48 and Tislelizumab for Renal Preservation in High-	RC48-ADC/Val-Cit/MMAE	HER2	2	2023

	risk Upper Urinary Tract Uroepithelial Carcinoma Patients				
55	Efficacy and Safety of Disitamab Vedotin Combined With Sintilimab in Second-line Treatment of Advanced Gastric Cancer: a Prospective, Single Arm Clinical Study (DVES)	RC48-ADC/Val-Cit/MMAE	HER2	2	2023
56	A single-arm, open-label, phase II study investigating anti-HER2 ADC plus anti-PD-1 antibody in patients with unresectable locally advanced or metastatic BTC with HER2 overexpression.	RC48-ADC/Val-Cit/MMAE	HER2	2	2023
57	An Open-label, Single Arm, Single Center Phase 2 Study of RC48-ADC (IV) in Combination With Gemcitabine(Intravesical) in High Risk NMIBC Subjects (BCG Naïve or BCG Unresponsive) That Expresses HER2 (IHC 1+ and Greater)	RC48-ADC/Val-Cit/MMAE	HER2	2	2023
58	An Open, Single Arm, Single Center Phase II Clinical Study Evaluating the Efficacy and Safety of RC48-ADC in the Treatment of Metastatic Castration Resistant Prostate Cancer (mCRPC) Progression After Novel Endocrine Therapy	RC48-ADC/Val-Cit/MMAE	HER2	2	2023
59	RC48 Combined With PD-1 and Radiotherapy as Bladdersparing Therapy in Patients With Muscular Infiltrating Bladder Uroepithelial Carcinoma With Limited HER-2 Expression Following Maximum Electrical Resection or Partial Cystectomy	RC48-ADC/Val-Cit/MMAE	HER2	2	2023
60	A Multicenter Phase II Study of RC48-ADC in Adjuvant/Salvage Treatment of HER2 Positive High-risk Non-muscle-invasive Bladder Cancer	RC48-ADC/Val-Cit/MMAE	HER2	2	2023
61	NeoAdjuvant Dynamic marker - Adjusted Personalized Therapy comparing trastuzumab-deruxtecan versus pacli-/docetaxel+carboplatin+trastuzumab+pertuzumab in HER2+ early breast cancer	DS-8201/Tetrapeptide-based/Topoisomerase I inhibitor	HER2	2	2023
62	KADCYLA AND NERATINIB FOR INTERCEPTION OF HER2+ BREAST CANCER WITH MOLECULAR RESIDUAL DISEASE	T-DM1/SMCC/DM1	HER2	2	2023
63	Disitamab Vedotin or Endocrine Therapy for Patients With Metastatic Breast Cancer With Hormone Receptor-positive and HER2-low-expression	RC48-ADC/Val-Cit/MMAE	HER2	3	2023
64	An Open-label, Randomized, Multi-center, Phase III Clinical Study of MRG002 Versus Investigator's Choice of Chemotherapy in	MRG002/Val-Cit/MMAE	HER2	3	2023

	the Treatment of Patients With HER2-positive Unresectable Locally Advanced or Metastatic Urothelial Cancer Previously Treated With Platinum-based Chemotherapy and PD-1/PD-L1 Inhibitors				
65	A Phase 3b, Multicenter, Global, Interventional, Open-label Study of Trastuzumab Deruxtecan (T-DXd), an Anti-HER2-Antibody Drug Conjugate (ADC), in Subjects Who Have Unresectable and/or Metastatic HER2-low or HER2 Immunohistochemistry (IHC) 0 Breast Cancer (DESTINY-Breast15)	DS-8201/tetrapeptide-based/topoisomerase I inhibitor	HER2	3	2023
66	A Study of RC48-ADC Combine With Toripalimab and Chemotherapy or RC48-ADC Combine With Toripalimab and Herceptin as First-line Treatment in Local Advanced or Metastatic Gastric Cancer With the HER2 Expression	RC48-ADC/Val-Cit/MMAE	HER2	3	2023
67	Disitamab Vedotin (RC48-ADC) in Patients With HER2-low Advanced Breast Cancer	RC48-ADC/Val-Cit/MMAE	HER2	Not Appli cable	2023
68	Disitamab Vedotin (RC48-ADC) in Patients With Breast Cancer: a Real World Study	RC48-ADC/Val-Cit/MMAE	HER2	Obser vatio nal	2023
69	A Phase I-II, First-in-Human Study of SKB264 in Patients With Locally Advanced Unresectable /Metastatic Solid Tumors Who Are Refractory to Available Standard Therapies	SKB264/sulfo-SPDB/topoisomerase I inhibitor	TROP-2	1,2	2020
70	A Phase I Study to Evaluate the Safety, Tolerability, Pharmacokinetics and Efficacy of FDA018-ADC in Patients With Advanced Solid Tumors	FDA018/Unknown/Unknown	TROP-2	1	2021
71	Phase 2, Open Label Study of DS-1062a, an Anti-TROP-2-Antibody-Drug Conjugate (ADC), in Patients With Advanced and/or Unresectable Non-Small Cell Lung Cancer (NSCLC), With Biomarker Analysis to Characterize Response to Therapy	DS-1062/Tetrapeptide-based/Topoisomerase I inhibitor	TROP-2	2	2021
72	TROPION-PanTumor01: Dose analysis of the TROP2-directed antibody-drug conjugate (ADC) DS-1062 (Dato-DXd, DS-1062) for the treatment (Tx) of advanced or metastatic non-small cell lung cancer (NSCLC).	DS-1062/Tetrapeptide-based/Topoisomerase I inhibitor	TROP-2	3	2021
73	A Phase II, Multicentre, Open-label, Master Protocol to Evaluate the Efficacy and Safety of DS-1062 (Dato-DXd) as Monotherapy and in Combination With Anticancer Agents in	DS-1062/Tetrapeptide-based/Topoisomerase I inhibitor	TROP-2	2	2022

	Patients With Advanced/Metastatic Solid Tumours (TROPION-PanTumor03)				
74	A Phase 3 Open-label, Randomised Study of DS-1062 (DatoDXd) With or Without Durvalumab Versus Investigator's Choice of Therapy in Patients With Stage I-III Triple-negative Breast Cancer Who Have Residual Invasive Disease in the Breast and/or Axillary Lymph Nodes at Surgical Resection Following Neoadjuvant Systemic Therapy (TROPION-Breast03)	DS-1062/Tetrapeptide-based/Topoisomerase I inhibitor	TROP-2	3	2022
75	A Phase 3, Open-label, Randomised Study of DS-1062 (Dato-DXd) Versus Investigator's Choice of Chemotherapy in Patients Who Are Not Candidates for PD-1/PD-L1 Inhibitor Therapy in First-line Locally Recurrent Inoperable or Metastatic Triple-negative Breast Cancer (TROPION Breast02)	DS-1062/Tetrapeptide-based/Topoisomerase I inhibitor	TROP-2	3	2022
76	A Phase III, Randomised, Open-label, Multicentre, Global Study of DS-1062 (Dato-DXd) in Combination With Durvalumab and Carboplatin Versus Pembrolizumab in Combination With Platinum-based Chemotherapy for the First-line Treatment of Patients With Locally Advanced or Metastatic NSCLC Without Actionable Genomic Alterations (D926NC00001; AVANZAR)	DS-1062/Tetrapeptide-based/Topoisomerase I inhibitor	TROP-2	3	2022
77	A Phase 3, Open-label, Randomized Study of Dato-DXd Versus Investigator's Choice of Chemotherapy in Participants With Inoperable or Metastatic Hormone Receptor-Positive, HER2-Negative Breast Cancer...	DS-1062/Tetrapeptide-based/Topoisomerase I inhibitor	TROP-2	3	2022
78	A Phase 1/2 Study to Evaluate the Safety, Tolerability, and Efficacy of TROP2-Directed Antibody-Drug Conjugate LCB84, as a Single Agent and in Combination With an Anti-PD-1 Ab, in Patients With Advanced Solid Tumors	DS-1062/Tetrapeptide-based/Topoisomerase I inhibitor	TROP-2	2	2023
79	An Open-label, Multicenter, Phase 3 Randomized, Active-Comparator-Controlled Clinical Study of Pembrolizumab (MK-3475) in Combination With Sacituzumab Govitecan Versus MK-3475 Monotherapy as First-line Treatment in Participants With PD L1 TPS Greater Than or Equal to 50% Metastatic Non-small Cell Lung Cancer (KEYNOTE D46/EVOKE-03)	IMMU-132/SMCC/Topoisomerase I inhibitor	TROP-2	3	2023

80	A Phase 1 Open-Label, Safety, Pharmacokinetic and Preliminary Efficacy Study of STRO-002, an Anti-Folate Receptor Alpha (FolRα) Antibody-Drug Conjugate (ADC), in Patients With Advanced Epithelial Ovarian Cancer (Including Fallopian Tube or Primary Peritoneal Cancers) and Endometrial Cancers	STRO-002/Val-Cit/Hemiasterlin	FRα	1	2019
81	A Multicenter, Open-Label Phase 1/2 Trial Evaluating the Safety, Tolerability, and Efficacy of MORAb-202, a Folate Receptor Alpha (FRα)-Targeting Antibody-drug Conjugate (ADC) in Subjects With Selected Tumor Types	MORAb-202/Val-Cit/Eribulin	FRα	1,2	2020
82	MIRASOL (GOG 3045/ENGOT OV-55): A randomized, open-label, phase III study of mirvetuximab soravtansine versus investigator's choice of chemotherapy in advanced high-grade epithelial ovarian, primary peritoneal, or fallopian tube cancers with high folate-alpha (FRα) expression.	IMGN-853/sulfo-SPDB/Maytansinoid DM4	FRα	3	2020
83	Randomized, Multicenter, Open-label, Phase 3 Study of Mirvetuximab Soravtansine in Combination With Bevacizumab Versus Bevacizumab Alone as Maintenance Therapy for Patients With FRα-high Recurrent Platinum-sensitive Epithelial Ovarian, Fallopian Tube, or Primary Peritoneal Cancers Who Have Not Progressed After Second Line Platinum-based Chemotherapy Plus Bevacizumab (GLORIOSA)	IMGN-853/sulfo-SPDB/Maytansinoid DM4	FRα	3	2022
84	FONTANA: A Modular Phase I/IIa, Open-label, Multi-center Study to Assess the Safety, Tolerability, Pharmacokinetics, and Preliminary Efficacy of Ascending Doses of AZD5335 Monotherapy and in Combination With Anti-cancer Agents in Participants With Solid Tumors	AZD5335/Unknown/Topoisomerase I inhibitor	FRα	1,2	2023
85	A Phase 2 Open-label Randomized Study of Farletuzumab Ecteribulin (MORAb-202), a Folate Receptor Alpha-targeting Antibody-drug Conjugate, Versus Investigator's Choice Chemotherapy in Women With Platinum-resistant High-grade Serous (HGS) Ovarian, Primary Peritoneal, or Fallopian Tube Cancer	MORAb-202/Val-Cit/Eribulin	FRα	2	2023
86	A Phase 2 Open-label Randomized Study of MORAb-202 (Farletuzumab Ecteribulin), a Folate Receptor Alpha-targeting Antibody-Drug Conjugate, in Participants with	MORAb-202/Val-Cit/Eribulin	FRα	2	2023

	Metastatic Non-Small Cell Lung Cancer ...				
87	A Phase 2 Open-label Randomized Study of Farletuzumab Ecteribulin (MORAb-202), a Folate Receptor Alpha-targeting Antibody-Drug Conjugate, versus Investigator's Choice Chemotherapy in Women with Pla...	MORAb-202/Val-Cit/Eribulin	FRα	2	2023
88	An Open-label, Single-arm, Multi-center, Phase II Clinical Study of MRG003 in the Treatment of Patients With EGFR-positive Unresectable, Locally Advanced or Metastatic Biliary Tract Cancer	MRG003/Val-Cit/MMAE	EGFR	2	2021
89	A Phase II Clinical Study to Evaluate the Efficacy and Safety of MRG003 in EGFR-Positive, HER2-Negative Advanced Gastric Cancer.	MRG003/Val-Cit/MMAE	EGFR	2	2021
90	An Open-Label, Multi-Center Phase II Clinical Study to Evaluate the Efficacy and Safety of MRG003 in Patients With Recurrent Metastatic Nasopharyngeal Carcinoma	MRG003/Val-Cit/MMAE	EGFR	2	2021
91	A Phase I/II First-in-human, Open Label, Multicenter, Dose Escalation and Cohort Expansion Study to Investigate the Safety, Tolerability, Pharmacokinetics and Preliminary Antitumor Activity of BB-1705 in Patients With Locally Advanced/Metastatic Solid Tumors	BB-1705/Val-Cit/Eribulin	EGFR	1,2	2022
92	An Open-label, Multi-center, Phase I/II Dose Escalation and Expansion Study to Evaluate the Safety, Tolerability, Pharmacokinetics and Efficacy of MRG003 in Combination With HX008 in Patients With EGFR-positive Advanced Solid Tumors	MRG003/Val-Cit/MMAE	EGFR	1,2	2022
93	BL-B01D1, a first-in-class EGFRxHER3 bispecific antibody-drug conjugate (ADC), in patients with locally advanced or metastatic solid tumor: Results from a first-in-human phase 1 study.	BL-B01D1/Val-Cit/Topoisomerase I inhibitor	EGFR	1	2023
94	A Randomized, Open-Label, Multicenter, Phase III Study to Evaluate MRG003 vs Cetuximab/Methotrexate as Second/Third Line of Treatment in Patient With Recurrent or Metastatic Squamous Cell Carcinoma of the Head and Neck (RM-SCCHN)	MRG003/Val-Cit/MMAE	EGFR	3	2023
95	A Phase I Study to Evaluate the Safety, Pharmacokinetics, and Effect of RC108-ADC For Injection in Subjects With c-Met Positive Advanced Malignant Solid Tumors	RC108/Unknown/Unknown	c-MET	1	2021
96	A First-in-human Dose-escalation and Expansion Trial With the Antibody-drug	BYON3521/Val-Cit/Duocarmacin	c-MET	1	2022

	Conjugate BYON3521 to Evaluate the Safety, Pharmacokinetics and Efficacy in Patients With c-MET Expressing Locally Advanced or Metastatic Solid Tumours				
97	A Study to Evaluate the Efficacy, Safety and Pharmacokinetics of RC108 for Injection in the Treatment of Patients With c-Met-positiveAdvanced Digestive System Malignant Tumor	RC108/Unknown/Unknown	c-MET	2	2022
98	A Phase 1 Multicenter Dose Escalation and Dose Expansion Study of Antibody-Drug Conjugate MYTX-011 in Subjects With Non-Small Cell Lung Cancer - KisMET-01	MYTX-011/Val-Cit/MMAE	c-MET	1	2023
99	A Dose-Escalation and Expansion Study of the Safety and Pharmacokinetics of XB002 as Single-Agent and Combination Therapy in Subjects With Inoperable Locally Advanced or Metastatic Solid Tumors	XB002/Val-Cit/MMAE	TF	1	2021
100	An Open-Label, Multi-center, Phase I/II Dose Escalation and Expansion Study to Assess the Safety, Tolerability, Anti-Tumor Activity and Pharmacokinetics of MRG004A in Patients With Tissue Factor Positive Advanced or Metastatic Solid Tumors	MRG004A/Unknown/Unknown	TF	1,2	2021
101	A Randomized, Open-Label, Phase 3 Trial of Tisotumab Vedotin Versus Investigator's Choice Chemotherapy in Second- or Third-Line Recurrent or Metastatic Cervical Cancer	TIVDAK/Val-Cit/MMAE	TF	3	2021
102	SWOG S1826: A Phase III, Randomized Study of Nivolumab Plus AVD or Brentuximab Vedotin Plus AVD in Patients with Newly Diagnosed Advanced Stage Classical Hodgkin Lymphoma	SGN35/Val-Cit/MMAE	TNFRSF8	3	2021
103	Brentuximab vedotin in combination with lenalidomide and rituximab in subjects with relapsed or refractory diffuse large B-cell lymphoma (DLBCL) (Trials in Progress).	SGN35/Val-Cit/MMAE	TNFRSF8	3	2021
104	798TiP Study EV-302: A 3-arm, open-label, randomized phase III study of enfortumab vedotin plus pembrolizumab and/or chemotherapy, versus chemotherapy alone, in untreated locally advanced or metastatic urothelial cancer	ASG-22ME/Val-Cit/MMAE	Nectin-4	3	2020
105	Nectin-4 Targeting ADC Probe for PET Imaging in Solid Tumors	PADCEV/Val-Cit/MMAE	Nectin-4	x	2023
106	Phase 2, Open Label Study of Patritumab Deruxtecan (U3-1402), an Anti-HER3-Antibody Drug Conjugate (ADC), in Patients With Advanced Breast Cancer, With	U3-1402/Tetrapeptide-based/Topoisomerase I inhibitor	HER3	2	2021

	Biomarker Analyses to Characterize Response to Therapy				
107	Results from the phase 1/2 study of patritumab deruxtecan, a HER3-directed antibody-drug conjugate (ADC), in patients with HER3-expressing metastatic breast cancer (MBC).	U3-1402/Tetrapeptide-based/Topoisomerase I inhibitor	HER3	1,2	2022
108	A Phase 1b, Multicenter, Two-Part, Open-Label Study of Trastuzumab Deruxtecan (DS-8201a), An Anti-Human Epidermal Growth Factor Receptor-2 (HER2)-Antibody Drug Conjugate (ADC), In Combination With Pembrolizumab, An Anti-PD-1 Antibody, For Subjects With Locally Advanced/Metastatic Breast Or Non-Small Cell Lung Cancer (NSCLC)	DS-8201/Tetrapeptide-based/Topoisomerase I inhibitor	ERBB2	1	2020
109	Antitumor Activity and Safety of Trastuzumab Deruxtecan in Patients With HER2-Low–Expressing Advanced Breast Cancer: Results From a Phase Ib Study	DS-8201/Tetrapeptide-based/Topoisomerase I inhibitor	ERBB2	1	2020
110	Phase III trial comparing antibody-drug conjugate (ADC) SAR408701 with docetaxel in patients with metastatic non-squamous non-small cell lung cancer (NSQ NSCLC) failing chemotherapy and immunotherapy.	SAR408701/SPDB/Maytansinoid DM4	CEACAM5	3	2020
111	A Phase I, Multicenter, Open-Label First in Human Study of Anti-CEACAM5 Antibody Drug Conjugate M9140 in Participants With Advanced Solid Tumors	M9140/Unknown/Topoisomerase I inhibitor	CEACAM5	1	2022
112	A Phase 1/ 2 Safety and Efficacy Dose Escalation / Dose Expansion Study of a CAB-AXL-ADC, Alone and in Combination With a PD-1 Inhibitor in Adult Patients With Advanced Solid Tumors (Phase 1) and Adult and Adolescent Patients With Advanced, Refractory Sarcoma (Phase 2)	CAB-AXL-ADC/Val-Cit/MMAE	AXL	1,2	2018
113	A Phase 2 Study of BA3011 Alone and in Combination With PD-1 Inhibitor in Adult Patients With Metastatic Non-small Cell Lung Cancer (NSCLC) Who Had Prior Disease Progression on a PD-1/L-1 Inhibitor	CAB-AXL-ADC/Val-Cit/MMAE	AXL	2	2021
114	A Phase 1/2 Safety and Efficacy Dose Escalation / Dose Expansion Study of a CAB-ROR2-ADC, Alone and in Combination With a PD-1 Inhibitor, in Patients With Advanced Solid Tumors (Ph1) and Melanoma and NSCLC Patients (Ph2)	CAB-ROR2-ADC/Val-Cit/MMAE	Ror2	1,2	2018
115	A Dose-Escalation Study to Evaluate the Safety, Tolerability, Pharmacokinetics, and	F0002-ADC/SMCC/Maytansinoid-DM1	CD30	1	2019

	Efficacy of F0002-ADC in Chinese Patients With Refractory or Recurrent CD30+ Hematologic Malignancies.				
116	A Phase I, Open-label, Dose Finding Study to Assess the Safety, Tolerability, PK, and Preliminary Efficacy of OBT076, a CD205-directed ADC, in Recurrent and/or Metastatic CD205+ Solid Tumors	OBT076/SPDP/Maytansinoid-DM4	CD205	1	2019
117	An Open-label, Multicenter, First-in-human, Phase I Dose-escalation and Expansion Clinical Study to Assess the Safety, Tolerability, Pharmacokinetics and Preliminary Efficacy of MRG001 in Patients With CD20-positive Relapsed or Refractory B-cell Non-Hodgkin Lymphoma (NHL)	MRG001/Val-Cit/MMAE	CD20	1	2019
118	A Phase 1/2, Open-Label, Dose-Escalation and Cohort-Expansion Study Evaluating the Safety, Pharmacokinetics, and Therapeutic Activity of OBI-999 in Patients With Advanced Solid Tumors	OBI-999/Unknown/MMAE	Globo H	1,2	2019
119	A phase I/II study of IMGN632, a novel CD123-targeting antibody-drug conjugate, in patients with relapsed/refractory acute myeloid leukemia, blastic plasmacytoid dendritic cell neoplasm, and other CD123-positive hematologic malignancies.	IMGN632/Val-Cit/IGN	CD123	1,2	2020
120	To Evaluate the Safety of RC88 for Injection in Patients With Advanced Malignant Solid Tumors,Multicenter, Open, Multi-cohort Extension of Efficacy and Pharmacokinetic Characteristics Phase I /IIa Clinical Study	RC88/Val-Cit/MMAE	Mesotheli n	1,2	2020
121	A phase II trial to assess the activity of Gemtuzumab Ozogamicin Therapy in Haemophagocytic lymphohistiocytosis (HLH) or Macrophage activation syndrome (MAS) or relapsed/refractory solid tumours	Gemtuzumab Ozogamicin/AcBut/Calicheamici n	CD33	2	2020
122	Sgnlva-005: Open-label, phase II study of ladiratuzumab vedotin (LV) for advanced aerodigestive tract malignancies.	SGN-LIV1A/Val-Cit/MMAE	LIV1	2	2020
123	Preliminary Results of a Phase 1 Dose Escalation Study of the First-in-Class Anti-CD74 Antibody Drug Conjugate (ADC), STRO-001, in Patients with Advanced B-Cell Malignancies	STRO-001/DBCO/Maytansinoid	CD74	1	2021
124	A Phase 1/2, Multicenter, Open-label, Dose-escalation, and Dose-expansion Study to Evaluate the Safety, Pharmacokinetics, and Anti-tumor Activity of ARX517, With Randomized Comparison to Investigator's	ARX517/Unknown/MMAF	PSMA	1,2	2021

	Choice of Treatment, in Subjects With Metastatic Castration-resistant Prostate Cancer Who Are Resistant or Refractory to Prior Standard Therapies				
125	ARTEMIS-001: A Phase 1, Open-label, Multi-center Study to Evaluate Safety, Tolerability, Pharmacokinetics, and Efficacy of Multiple Doses of Intravenous Administration of HS-20093 in Patients With Locally Advanced or Metastatic Solid Tumors Who Have Progressed Following Prior Therapy	HS-20093/Unknown/Unknown	B7-H3	1	2021
126	A Phase 1, First in Human, Dose-Escalation Study of TORL-2-307-ADC in Participants With Advanced Cancer	TORL-2-307-ADC/Unknown/MMAE	CLDN 18.2	1	2022
127	A Phase I, Open-label, Multicenter Study to Evaluate Safety, Tolerability, Pharmacokinetics, and Efficacy of HS-20089 in Patients With Advanced Solid Tumors	HS-20089/Unknown/Unknown	B7-H4	1	2022
128	A Phase 3, Randomized, Double-blind, Placebo-controlled, Multicenter Study of Upifitamab Rilsodotin (XMT-1536) as Post-Platinum Maintenance Therapy for Participants With Recurrent, Platinum-Sensitive, Ovarian Cancer (UP-NEXT)	XMT-1536/Thioether bond/auristatin F	NaPi2b	3	2023
